# Supplementary material for: GWAS, MWAS and mGWAS provide insights into precision agriculture based on genotype-dependent microbial effects in foxtail millet
Source: Nat Commun. 2022 Oct 7;13:5913. doi: 10.1038/s41467-022-33238-4 (PMC9546826; doi:10.1038/s41467-022-33238-4)
Supplement: Supplementary file 1 — Supplementary information [file 41467_2022_33238_MOESM1_ESM.pdf]

GWAS, MWAS and mGWAS provide insights into precision agriculture based on genotype-dependent microbial effects in foxtail millet

Wang *et al.*

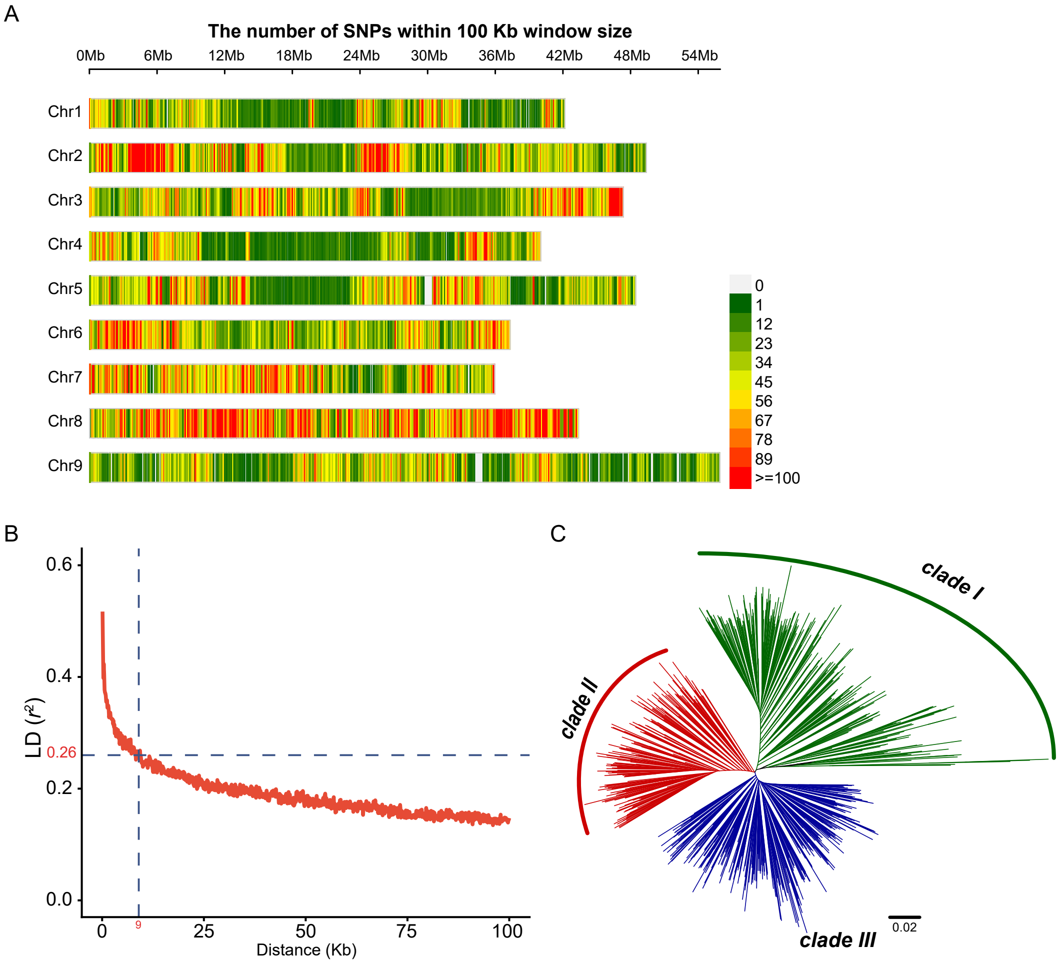

Supplementary Figure 1. The genetic structures of 827 foxtail millet varieties. (A) The SNPs distributed along the chromosome show the linkage equilibrium. (B) The plot of genome-wide linkage disequilibrium (LD) decay. The X-axis represents the genetic distance (Kb) between SNPs and the y-axis represents LD value (r^2). Horizontal and vertical lines represent half LD and LD decay distance respectively, and LD decays to its half decay at the distance of 9 kb. (**C**) The phylogenic tree is constructed based on genetic SNPs. All the cultivars are divided into three groups.

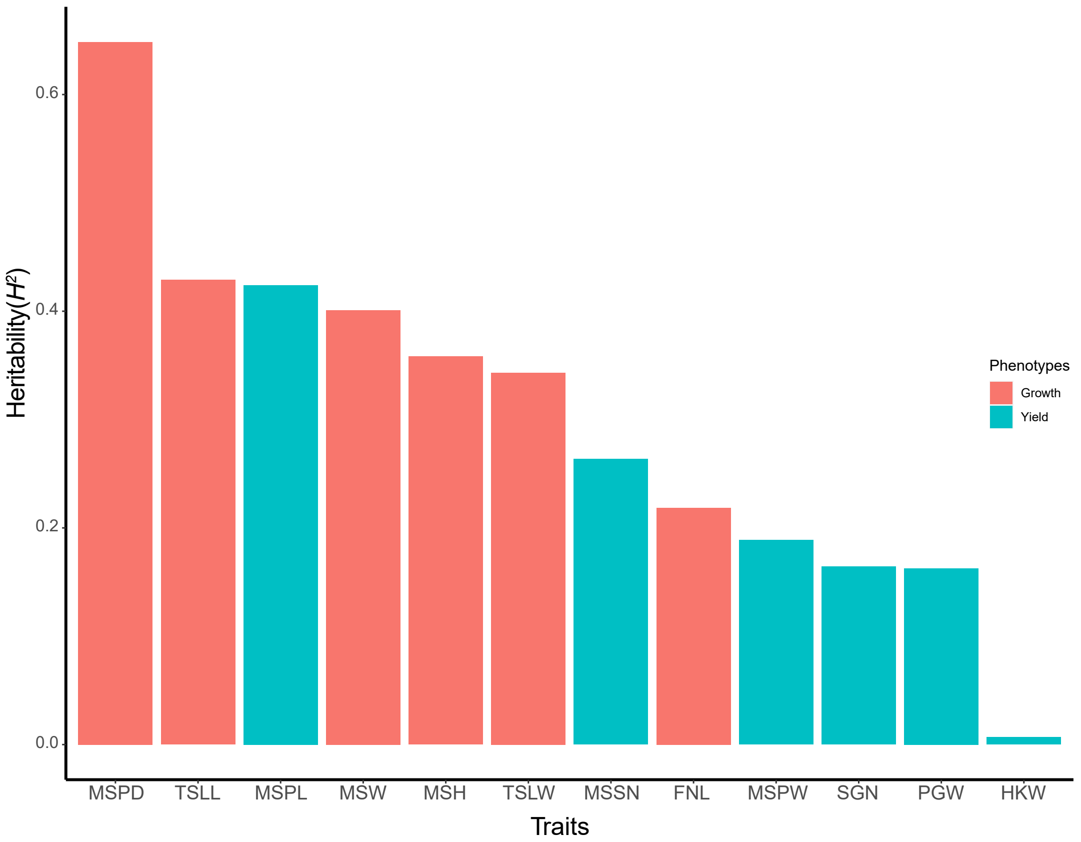

Supplementary Figure 2. Broad-sense heritability of 12 agronomic traits for the foxtail millet in the field study. The adjusted *P* for most of the traits is less than 0.05 except HKW. Six growth traits are marked with red colour, namely top second leaf length (TSLL), top second leaf width (TSLW), main stem height (MSH), main stem width (MSW), panicle diameter of the main stem (MSPD) and fringe neck length (FNL), and six yield traits marked with green colour, namely panicle length of the main stem (MSPL), panicle grain weight per plant (PGW), panicle weight of main stem (MSPW), hundred kernel weight (HKW), spikelet number of the main stem(MSSN) and grain number per spike (SGN).

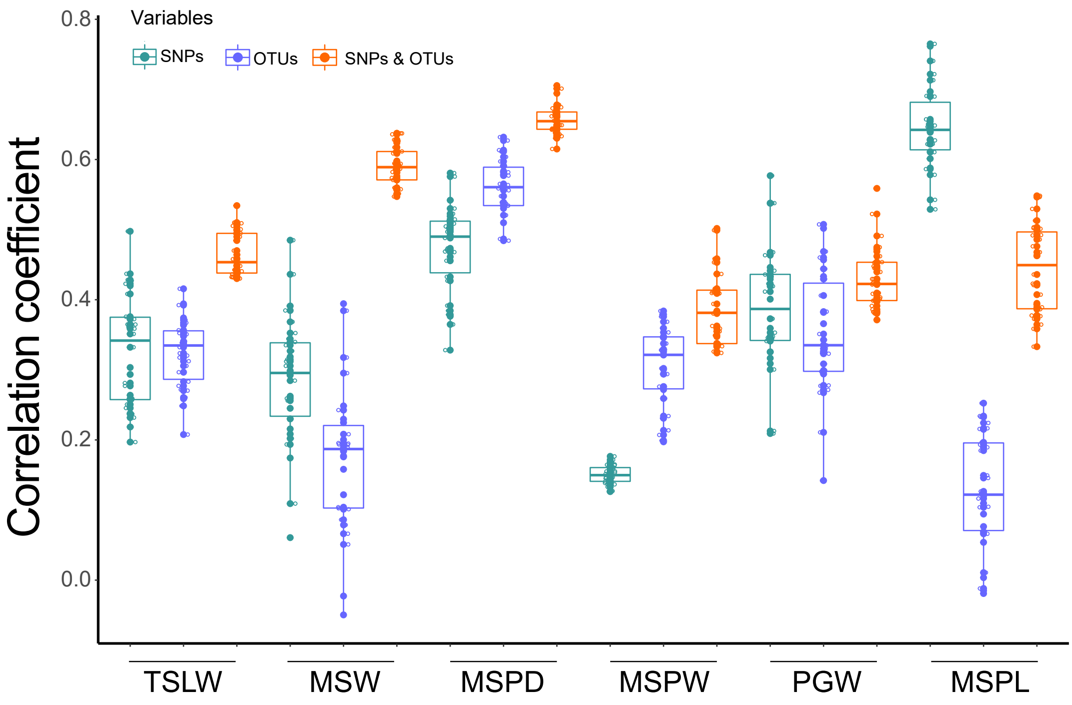

Supplementary Figure 3. The R^2 between the observations and predictions of traits in the testing dataset from thirty different repeats of five-fold cross validation. The sample number in the testing dataset of TSLW, MSW, MSPD, MSPW, PGW and MSPL are 136, 100, 117, 126, 110, and 106, respectively. The variation of the trait is predicted by genetic SNPs alone (Green dots), rhizoplane OTUs alone (blue dots) or by host genetic SNPs and rhizoplane OTUs combined (orange dots), respectively. The box edge depicts the 75th and 25th percentiles, respectively and the line within the box represents the median. The whiskers extend to the minimum and maximum data values.

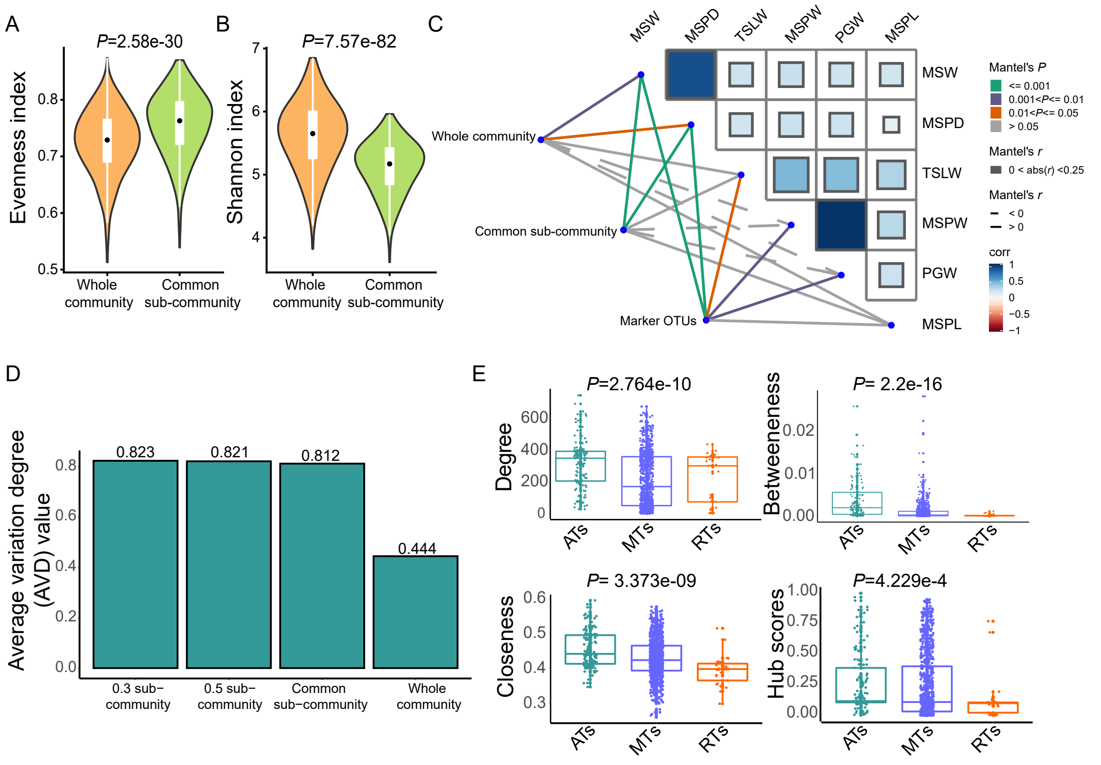

Supplementary Figure 4. The structure of a whole microbial community and common sub-community in the rhizoplane of foxtail millet. The violin chart displays the alpha diversities of the whole community (14869 OTUs) and common sub-community (1004 OTUs) in rhizoplane, including evenness (A) and Shannon index (B). The common sub-community have higher evenness but less diversity than the whole microbial community. The significant difference is determined by the Kruskal-Wallis test (one-way) and the *P*-value (*P*) is shown on the top of the panel. n=827 for both plots A and B. (C) the correlations of different microbial communities (whole, common and marker) to each trait are detected by Mantel tests. Edge width corresponds to the correlations of Mantel's *r* statistic, and edge color denotes the statistical significance based on 999 permutations. (D) the barplot displays the average variation degree (AVD) value of different microbial communities, such as 0.3 sub-community, 0.5 sub-community, common sub-community and the whole community. (E) topological features (degree, closeness, betweenness centrality and hub scores) of the co-occurrence network are compared among abundant OTUs (ATs, n=129), moderate OTUs (MTs, n=840) and rare OTUs (RTs, n=35) of common sub-community. The significant difference was determined by Kruskal-Wallis test (one-way). The box edge depicts the 75th and 25th percentiles, respectively and the line within the box represents the median. The whiskers extend to the minimum and maximum data values.

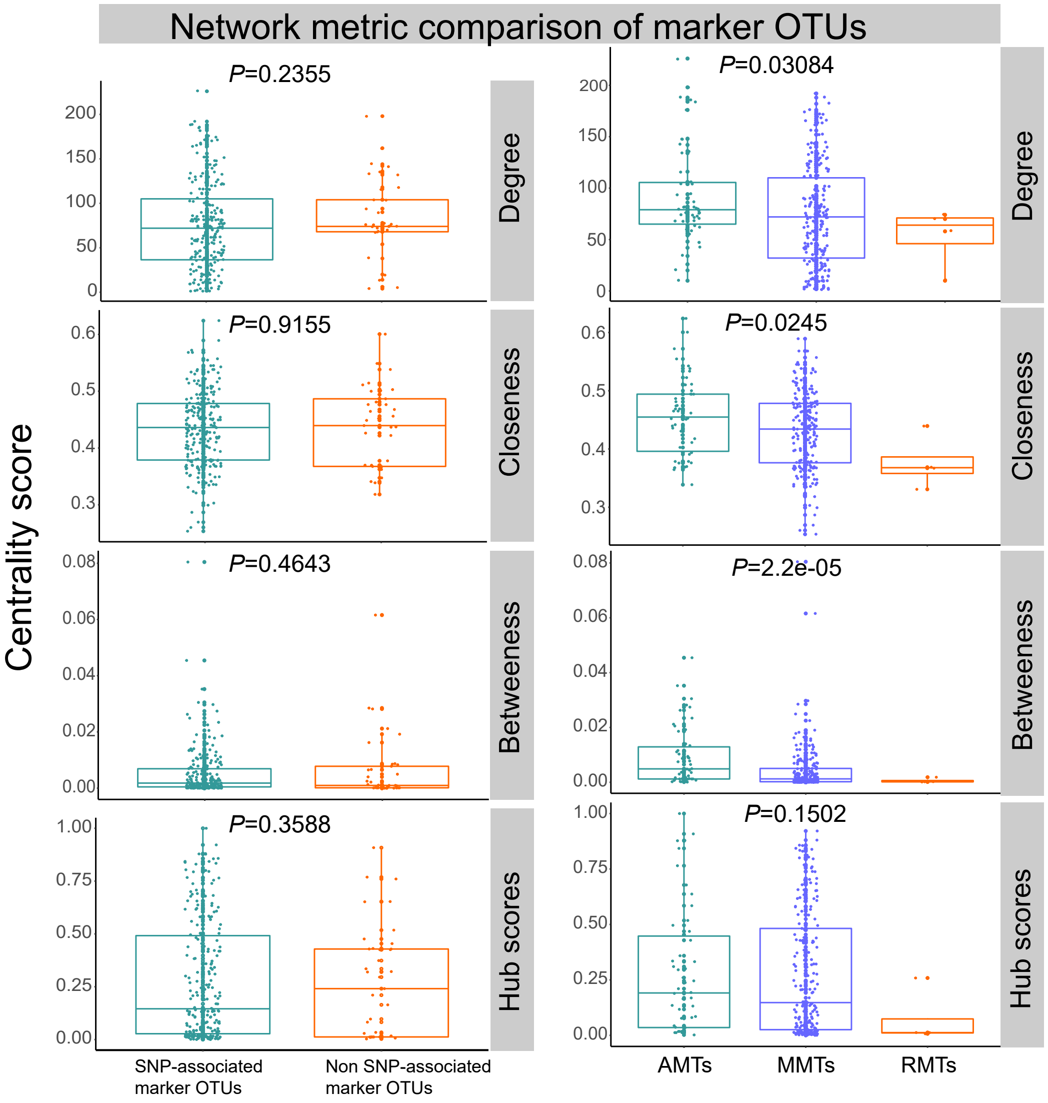

Supplementary Figure 5. (A) topological features (degree, closeness, betweenness centrality and hub scores) have no differences between the co-occurrence network of SNP-associated marker OTUs (n=219) and non SNP-associated marker OTUs (n=39). (B) the degree, closeness and betweenness centrality differed among the abundant marker OUTs (AMTs, n=50), moderate marker OTUs (MMTs, n=203) and rare marker OTUs (RMTs, n=4). The significant difference is determined by Kruskal-Wallis one-way analysis and the *P*-value (*P*) is shown on the top of the panel. The box depicts the interquartile range (IQR) between the 25th and 75th percentiles, respectively and the line within the box represents the median. The whiskers extend 1.5 times the IQR from the top and bottom of the box.

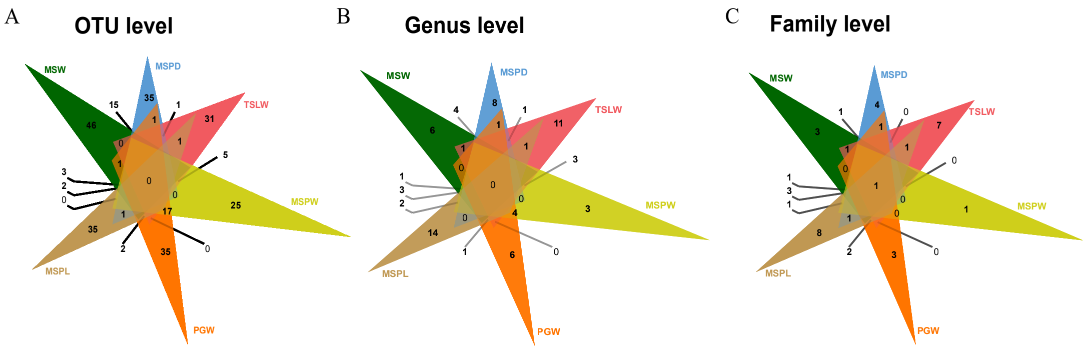

Supplementary Figure 6. The Venn diagram displays the overlap of microbial markers among six traits at OTUs level (A), genus level (B) and family level (C), respectively. Only one family Chitinophagaceae was shared by all the six traits, while neither OTU nor genus was shared by all six traits.

A

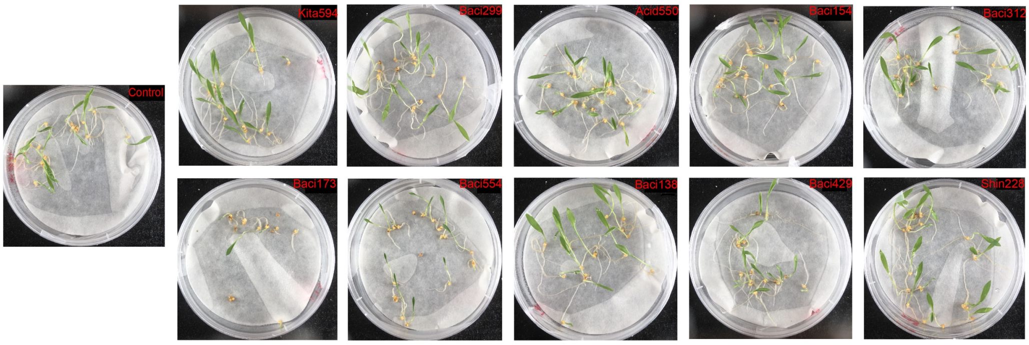

B

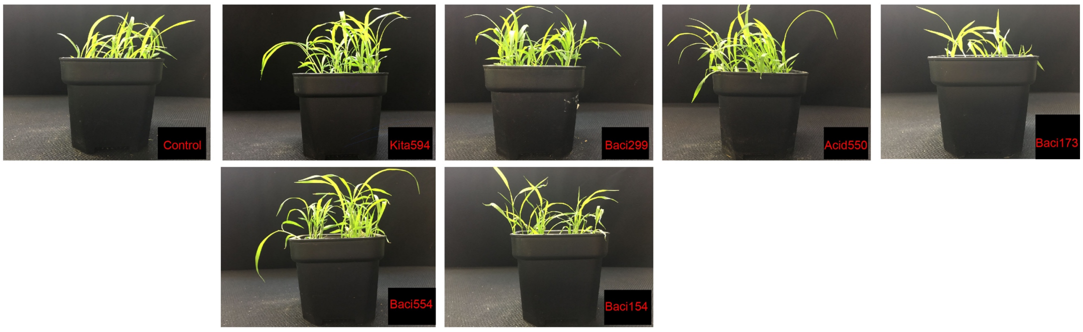

Supplementary Figure 7. The promoting and suppressing effect of marker microbes on foxtail millet Huagu12. (A) the plant height and root length of the Huagu12 after inoculating marker stains for 7 days in a sterilized plate. (**B**) the picture of Huagu12 after inoculating the marker stains for 14 days in sterilized soil. The pictures were taken by Yayu Wang.

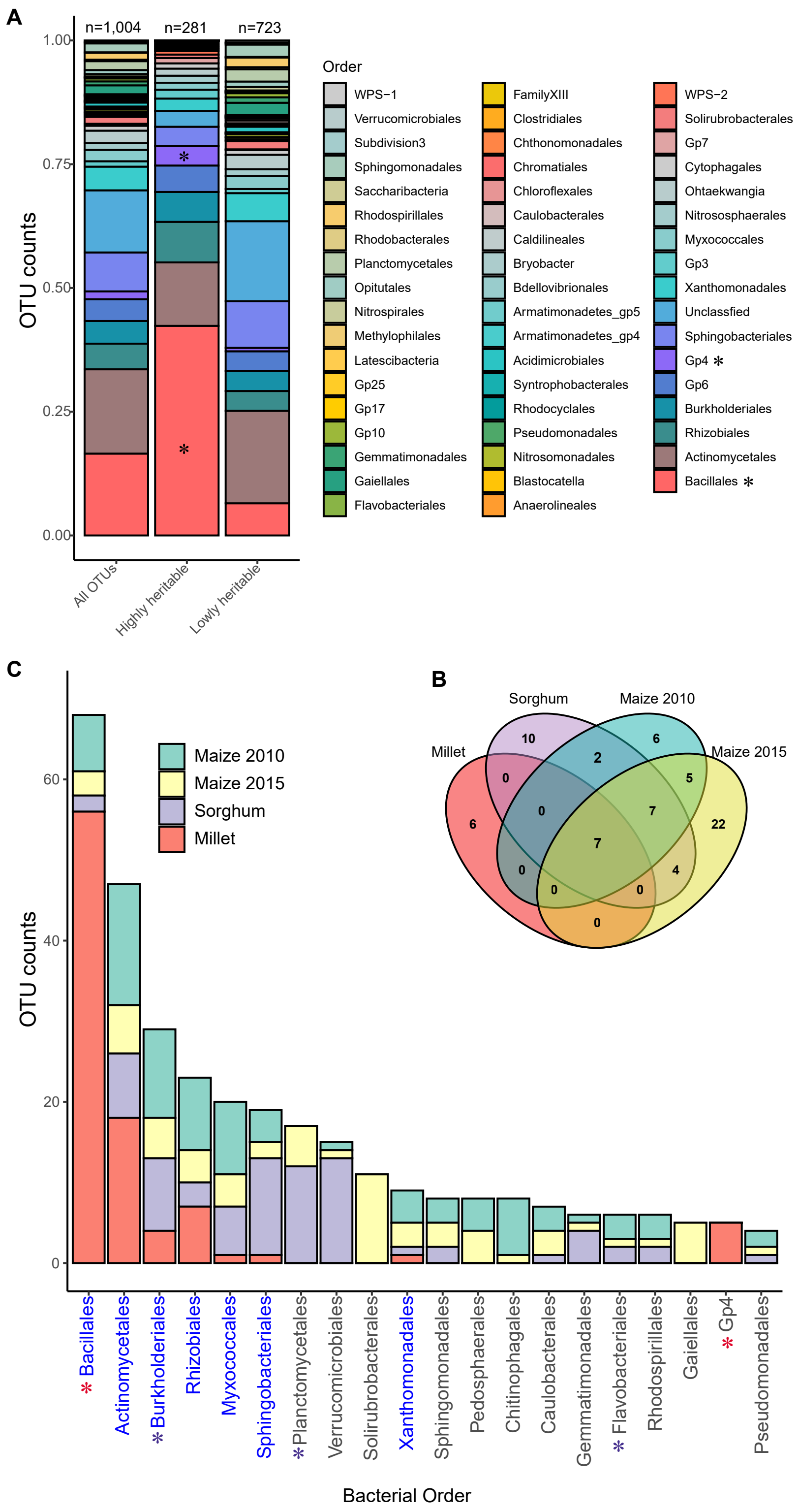

Supplementary Figure 8. Broad-sense heritability of individual OTUs for foxtail millet. (A) The numbers of microbial OTUs belong to each bacterial order for all common OTUs (left bar), highly heritable OTUs or low heritable OTUs. Orders with higher numbers of OTUs in the highly heritable (H^2>0.15) as compared to the lowly heritable fraction (H^2<0.15) are determined by one-sided Fisher's exact test (q <0.05), and are indicated with black asterisks. (B) the barplots displayed the OTUs counts of

the top 100 highly heritable OTUs in any four datasets. Each order has a total of at least four highly heritable OTUs. C. the top 100 heritable OTUs from each dataset are classified at order level to generate the Venn diagram. Seven orders (blue) are shared by all four datasets. The red asterisks indicate the orders with higher numbers of highly heritable OTUs in the foxtail millet dataset while the blue asterisks represent the orders with higher numbers of highly heritable OTUs in the sorghum dataset.

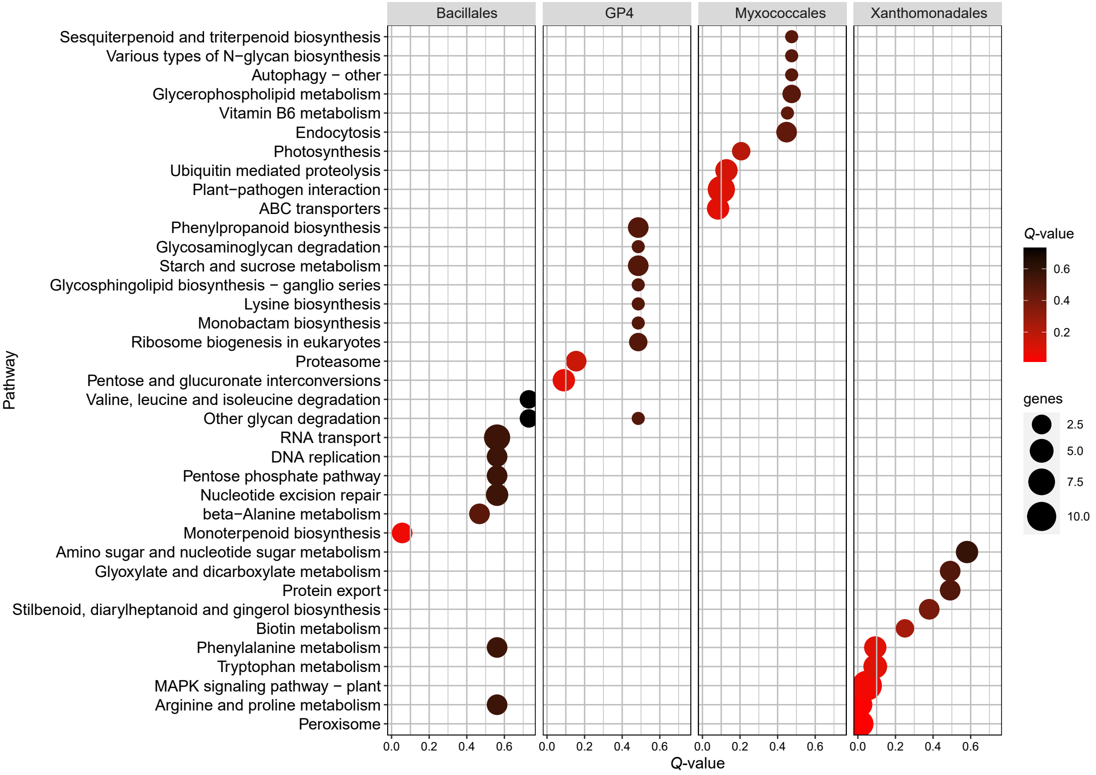

Supplementary Figure 9. KEGG enrichment analysis of the biological functions and processes that underlie interactions between the host genome and heritable bacteria, especially the orders more sensitive to genotypes. Only the orders with pathways that *q*-value <0.1 are displayed.

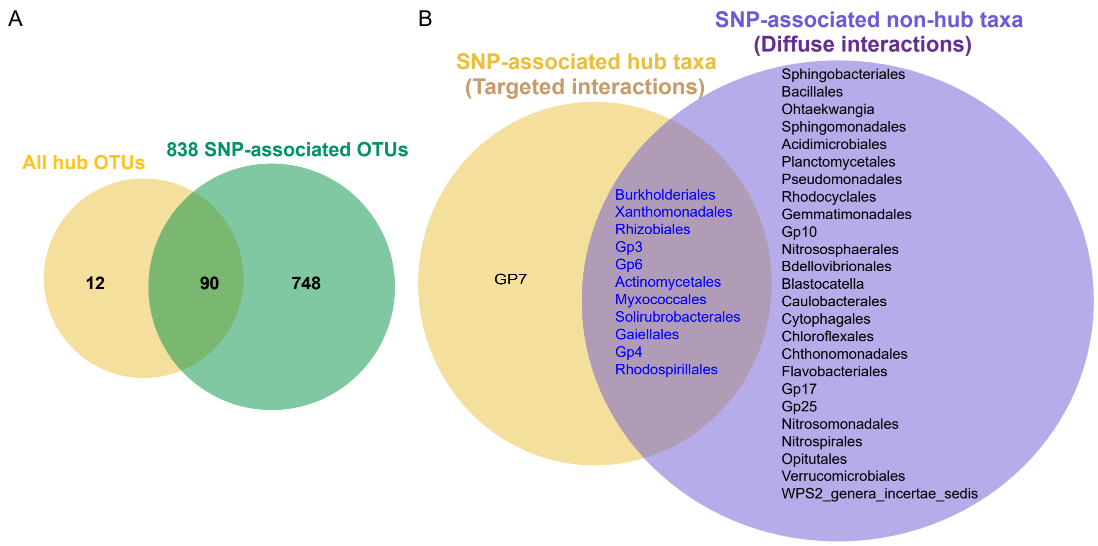

Supplementary Figure 10. Characterization of the genotype-associated hub and non-hub OTUs in the rhizoplane microbiota. A Venn diagram depicting the numbers of SNP-associated hub OTUs and non-hub OTUs. B Venn diagram depicting the shared and specific taxa between the SNP-associated hub and non-hub tax at the order level.

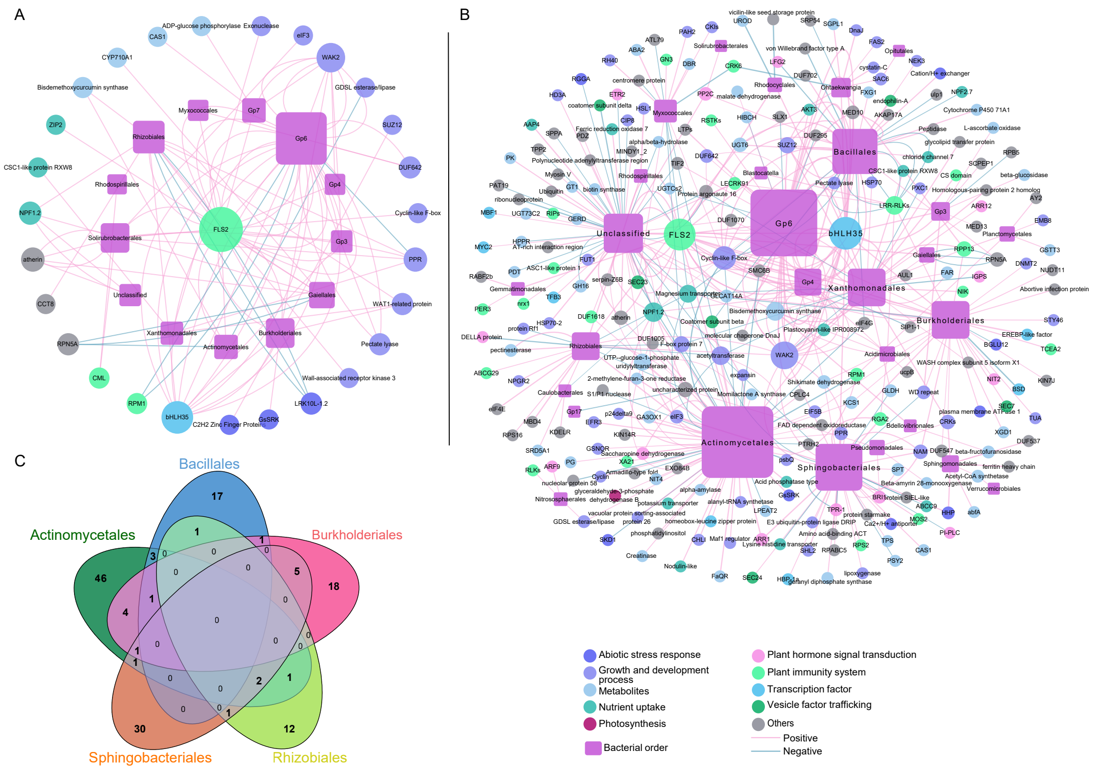

Supplementary Figure 11. The association networks of host genes with hub taxa (A) and non-hub taxa (B). (C) Venn diagram depicting the differences of the associating host genes among the different non-hub taxa.

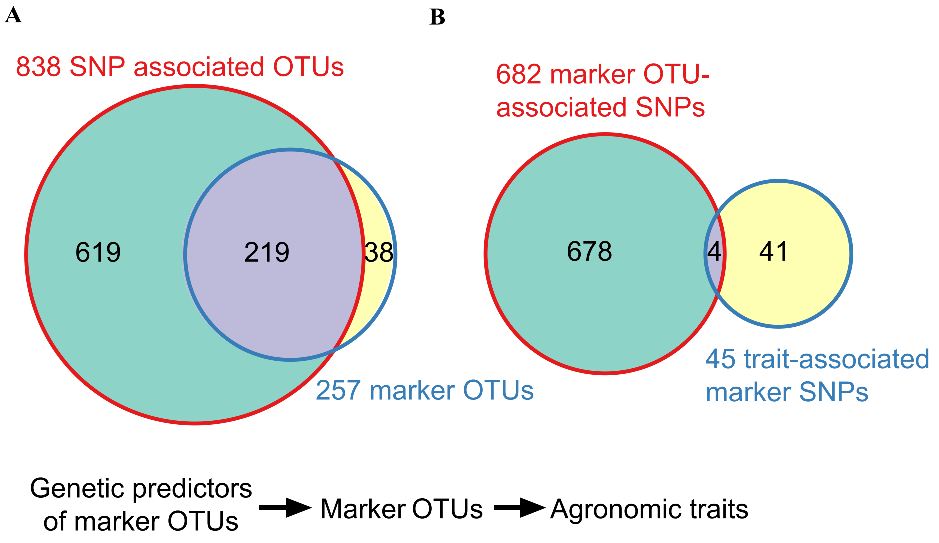

Supplementary Figure 12. Venn diagrams showing the substantial overlap between the marker OTUs and the SNP-associated OTUs (A), whereas rarely overlap between marker OTU associated SNPs and marker SNPs associated with traits (B). The schematic representation of a model in which host genetic variation influences root microbiome composition to modulate agronomic traits are shown.

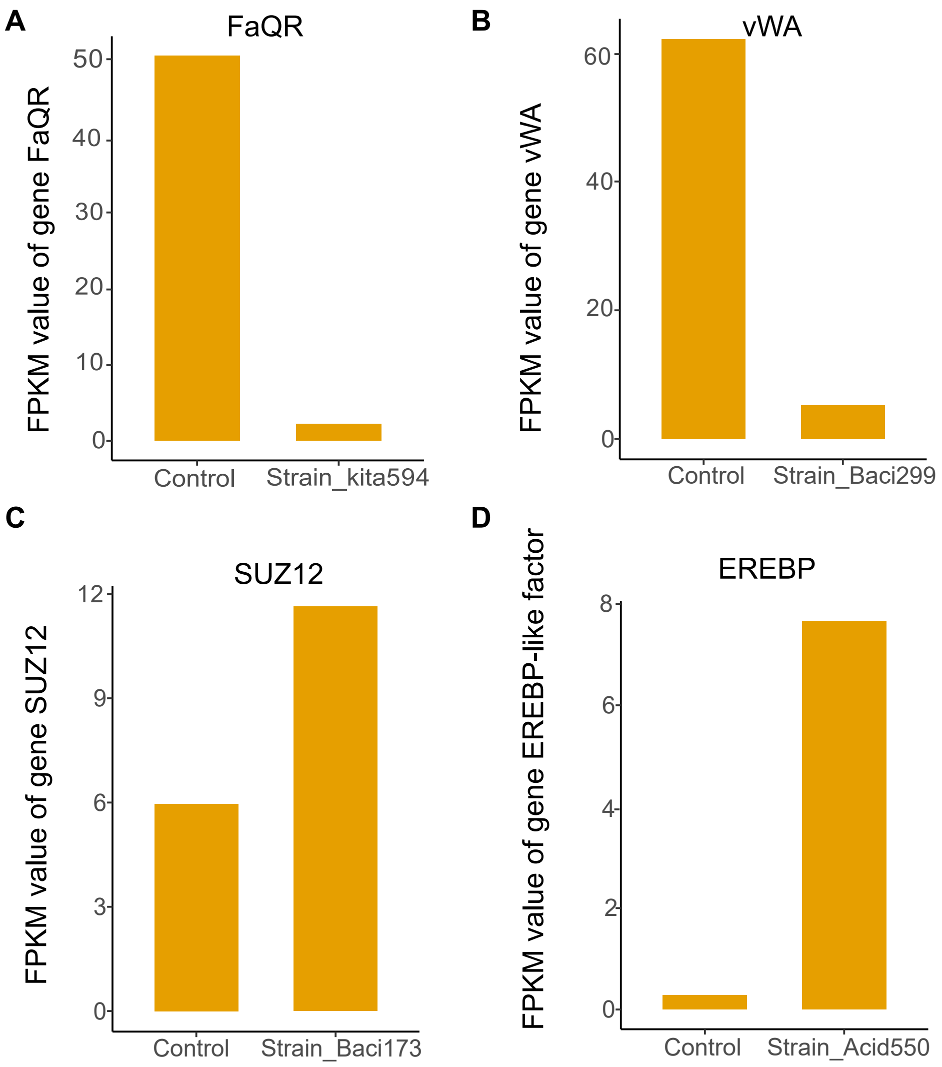

Supplementary Figure 13. The expression pattern of host candidate genes varied after inoculating marker strains in sterilized soil. (A-B) The expressions of host genes FaQR (2-methylene-furan-3-one reductase) and vWA (von Willebrand factor, type A) are significantly down-regulated after inoculating marker strain Kita594 (OTU_8), and Baci299 (OTU_22228) compared to control, respectively. (C-D) The expressions of host gene SUZ12 (Polycomb protein SUZ12) and EREBP-like factor (ethylene response element binding protein) are significantly up-regulated after inoculating marker strain Baci173 (OTU_19835) and Acid550 (OTU_46) compare to control, respectively.

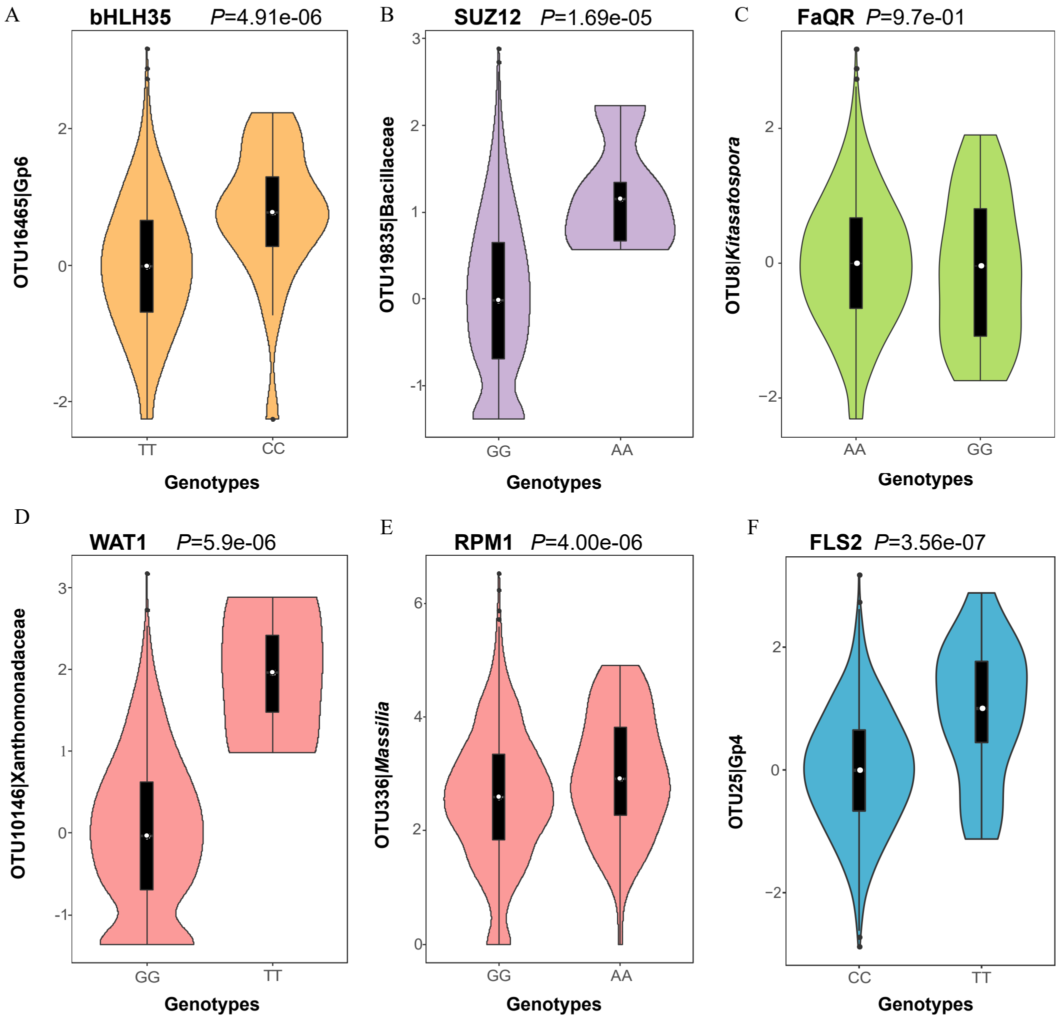

Supplementary Figure 14. The abundance of the marker microbial OTUs shifts between the genotypes of the associated genes. (A-F) The violin plots show the normalized abundances of marker OTUs within each genotype at the most strongly associated SNP and the *P*-values of the SNPs on the top of the panel. The left bar plot shows the abundance of marker OTU in reference genotype cultivars and the right bar plot shows its abundance in allele genotype cultivars. The significant difference in the abundance between different genotype cultivars is detected by Wilcox rank sum test. (A)OTU16465|GP6 (*P*=0.0002). (B) OTU_19835|Bacillaceae (*P*= 0.0001). (C)OTU_8| *Kitasatospora* (*P*= 0.2953). (D) OTU10146|Xanthomonadaceae (*P*= 0.004). (E) OTU336|*Massilia* (*P*=0.002). (F) OTU25|Gp4 (*P*= 0.0003). The sample number of reference genotype cultivars and the allele genotype cultivars in plots A-F from left to right is 782 to 21, 800 to 9, 813 to 8, 801 to 3, 678 to 69, and 799 to 16. Each functional gene is abbreviated as follows: bHLH35, Basic helix-loop-helix protein 35; SUZ12, Polycomb protein SUZ12; FaQR, 2-methylene-furan-3-one reductase; WAT1, Protein WALLS ARE THIN 1; RPM1, Disease resistance protein RPM1; FLS2, LRR receptor-like serine/threonine-protein kinase FLS2. Box plots show the median and the 25% and 75% quantiles.

Supplementary Table 1. The OTU distributions of the whole microbial community, common sub-community and marker OTUs. The OTUs with relative abundances ≥0.1% across more than 50% of samples are defined as abundant OTUs (AT), whereas the OTUs with relative abundances <0.01% across more than 30% of samples but never abundant (≥0.1%) more than 30% samples are defined as rare OTUs (MT). Those OTUs neither belonging to abundant taxa nor rare taxa are defined as moderate OTUs (MT).

**Category**	**Abundant OTUs (ATs)**	**Moderate OTUs (MTs)**	**Rare OTUs (RTs)**
	OTU numbers (percentage)	OTU numbers (percentage)	OTU numbers (percentage)
Whole community (14869 OTUs)	129(0.87%)	855(5.75%)	13885 (93.38%)
Common sub-community (1004 OTUS)	129(12.85%)	840(83.67%)	35(3.48%)
Marker OTUs (257 OTUs)	50(19.45%)	203 (78.99%)	4(1.56%)

Supplementary Table 2 The correlations of SNP-associated marker OTUs and non SNP-associated marker OTUs with six growth and yield traits are calculated with Mantel test, separately.

**Item**	**OTUs**	**OTU numbers**	**Phenotype**	**Mantel r**	***P*-value**
TSLW	All marker OTUs	40	TSLW	0.0488	0.012
	SNP-associated marker OTUs	36	TSLW	0.0446	0.022
	Non SNP-associated marker OTUs	4	TSLW	0.0518	0.004
MSPD	All marker OTUs	54	MSPD	0.1962	0.001
	SNP-associated marker OTUs	45	MSPD	0.2155	0.001
	Non SNP-associated marker OTUs	9	MSPD	0.0764	0.001
MSW	All marker OTUs	70	MSW	0.1893	0.001
	SNP-associated marker OTUs	60	MSW	0.215	0.001
	Non SNP-associated marker OTUs	10	MSW	0.0348	0.023
MSPW	All marker OTUs	50	MSPW	0.05	0.008
	SNP-associated marker OTUs	40	MSPW	0.0504	0.007
	Non SNP-associated marker OTUs	10	MSPW	0.0352	0.027
PGW	All marker OTUs	56	PGW	0.0592	0.002
	SNP-associated marker OTUs	48	PGW	0.0611	0.002
	Non SNP-associated marker OTUs	8	PGW	0.0285	0.05
MSPL	All marker OTUs	43	MSPL	0.0298	0.079
	SNP-associated marker OTUs	37	MSPL	0.026	0.116
	Non SNP-associated marker OTUs	6	MSPL	0.0389	0.022

Supplementary Table 3. The effects of genotypes and strains (Baci173 and Kita594) on foxtail millet growth were detected by PERMANOVA. *, ** and *** represented the $P < 0.05$, 0.01 and 0.001, respectively.

Call: adonis(formula = Root length ~ Genotypes * Strain, data = strain173, permutations =999)							
	Df	SumsOfSqs	MeanSqs	F.Model	R2	Pr(>F)	
Genotypes	1	0.0331	0.03312	1.949	0.00515	0.153	
Strain	1	0.7807	0.78073	45.957	0.1214	0.001	***
Genotypes:Strain	1	0.4867	0.48671	28.649	0.07568	0.001	***
Residuals	302	5.1305	0.01699	0.79777			
Total	305	6.4311	1				
Call: adonis(formula = Height ~ Genotypes * Strain, data = strain173, permutations =999)							
	Df	SumsOfSqs	MeanSqs	F.Model	R2	Pr(>F)	
Genotypes	1	0.1001	0.100104	6.0666	0.01891	0.016	*
Strain	1	0.0711	0.071119	4.31	0.01344	0.049	*
Genotypes:Strain	1	0.1383	0.13833	8.3833	0.02614	0.003	**
Residuals	302	4.9832	0.016501	0.94151			
Total	305	5.2928	1				
Call: adonis(formula = Root length ~ Genotypes * Strain, data = strain594, permutations =999)							
	Df	SumsOfSqs	MeanSqs	F.Model	R2	Pr(>F)	
Genotypes	1	0.1048	0.104774	5.3369	0.01729	0.012	*
Strain	1	0.0468	0.046788	2.3833	0.00772	0.113	
Genotypes:Strain	1	0.04	0.039959	2.0354	0.00659	0.118	
Residuals	299	5.87	0.019632	0.9684			
Total	302	6.0616	1				
Call: adonis(formula = Height ~ Genotypes * Strain, data = strain594, permutations = 999)							
	Df	SumsOfSqs	MeanSqs	F.Model	R2	Pr(>F)	
Genotypes	1	1.1762	1.1762	65.433	0.16594	0.001	***
Strain	1	0.3024	0.30242	16.824	0.04267	0.001	***
Genotypes:Strain	1	0.2345	0.23454	13.048	0.03309	0.001	***
Residuals	299	5.3748	0.01798	0.7583			
Total	302	7.0879	1				

Supplementary Table 4. The primer pair is used to amplify the 16S rRNA gene of the bacterial strain.

**Primers for full length of 16S rRNA gene**	**Primer name**	**Primer sequences**
Forward primer	27F	5'-GAGTTTGATCCTGGCTCAG-3'
Reverse primer	1492R	5'-TACCTTGTTACGACTT-3'
