# Supplementary material for: GWAS, MWAS and mGWAS provide insights into precision agriculture based on genotype-dependent microbial effects in foxtail millet
Source: Nat Commun. 2022 Oct 7;13:5913. doi: 10.1038/s41467-022-33238-4 (PMC9546826; doi:10.1038/s41467-022-33238-4)
Supplement: Supplementary file 2 — Description of Additional Supplementary Files [file 41467_2022_33238_MOESM2_ESM.pdf]

Description of Additional Supplementary Files

File Name: Supplementary Data 1
Description: The detail of 12 traits were collected from foxtail millet planted in Yangling, China.

File Name: Supplementary Data 2
Description: The genetic SNPs associated with 12 phenotypes under suggestive *P*-value thresholds (adjusted $P < 2.01e-5$, two-tailed test)

File Name: Supplementary Data 3
Description: The candidate genes associated with significant SNPs for foxtail millet traits.

File Name: Supplementary Data 4
Description: The genetic SNPs associated with growth (MSPD, MSW, TSLW) and yield trait (MSPW, PGW, MSPL) in the linear mixed model, respectively. The candidate SNPs for each trait are selected with an adjusted *P*-value < 1.0e-4. The marker SNPs in the best predictive model with the Pr(>|t|) are listed in the table.

File Name: Supplementary Data 5
Description: The microbial OTUs associated with growth (MSPD, MSW, TSLW) and yield trait (MSPW, PGW, MSPL), respectively. The candidate OTUs for each trait are selected with an adjusted *P*-value <0.05. The marker OTUs in the best predictive model with the Pr(>|t|) are listed in the table.

File Name: Supplementary Data 6
Description: The information of 257 bacterial isolations.

File Name: Supplementary Data 7
Description: The relative abundance of the strain-specific induction of gene.

File Name: Supplementary Data 8
Description: The heritability of 1004 common OTUs of foxtail millet.

File Name: Supplementary Data 9
Description: The details for the associations between the candidate genes from foxtail millet and root microbial OTUs.

File Name: Supplementary Data 10
Description: The enriched pathways of host plant genes associated with the marker OTUs. The *P*-value of the enrichment of a pathway is calculated using a One-sided Fisher's exact test and multiple testing is corrected using the Benjamini-Hochberg (BH) procedure.
