# Supplementary material for: GWAS, MWAS and mGWAS provide insights into precision agriculture based on genotype-dependent microbial effects in foxtail millet
Source: Nat Commun. 2022 Oct 7;13:5913. doi: 10.1038/s41467-022-33238-4 (PMC9546826; doi:10.1038/s41467-022-33238-4)
Supplement: Supplementary file 3 — Reporting Summary [file 41467_2022_33238_MOESM3_ESM.pdf]

nature portfolio

Corresponding author(s): Ertao Wang
Huan Liu

Last updated by author(s): Aug 24, 2022

Reporting Summary

Nature Portfolio wishes to improve the reproducibility of the work that we publish. This form provides structure for consistency and transparency in reporting. For further information on Nature Portfolio policies, see our Editorial Policies and the Editorial Policy Checklist.

Statistics

For all statistical analyses, confirm that the following items are present in the figure legend, table legend, main text, or Methods section.

n/a	Confirmed	
☐	☒	The exact sample size (*n*) for each experimental group/condition, given as a discrete number and unit of measurement
☐	☒	A statement on whether measurements were taken from distinct samples or whether the same sample was measured repeatedly
☐	☒	The statistical test(s) used AND whether they are one- or two-sided<br>*Only common tests should be described solely by name; describe more complex techniques in the Methods section.*
☒	☐	A description of all covariates tested
☐	☒	A description of any assumptions or corrections, such as tests of normality and adjustment for multiple comparisons
☐	☒	A full description of the statistical parameters including central tendency (e.g. means) or other basic estimates (e.g. regression coefficient) AND variation (e.g. standard deviation) or associated estimates of uncertainty (e.g. confidence intervals)
☐	☒	For null hypothesis testing, the test statistic (e.g. *F*, *t*, *r*) with confidence intervals, effect sizes, degrees of freedom and *P* value noted<br>*Give P values as exact values whenever suitable.*
☒	☐	For Bayesian analysis, information on the choice of priors and Markov chain Monte Carlo settings
☒	☐	For hierarchical and complex designs, identification of the appropriate level for tests and full reporting of outcomes
☒	☐	Estimates of effect sizes (e.g. Cohen's *d*, Pearson's *r*), indicating how they were calculated

Our web collection on statistics for biologists contains articles on many of the points above.

Software and code

Policy information about availability of computer code

Data collection	No software was used for data collection.
Data analysis	The list of Software used in this study are as follows:<br>SOAPnuke (v.1.5.6), BWA-MEM (v. 0.7.12-r1039), SAMtools (v.0.1.19-44428cd), Picard (v.1.54), GATK (v.3.6), BCFtools (v.1.2), BEAGLE (v.4.1), PLINK (v.1.90), snpEff (v.4.3t), GEMMA (v.0.98), EnrichmentPipeline (v.1.01),R packages igraph 1.2.11, R package lsr 0.5.2, GCTA(v.1.92.2), Usearch (v.10.0.240), SOAPdenovo(v.2.0), hisat2 (v.2.0.4), Subread (v.1.6.4), ballgown (v.2.22.0), DEseq (v.1.38.0) and R package(v.4.0.2).<br>The source code are publicly available at https://zenodo.org/badge/latestdoi/424864991.

For manuscripts utilizing custom algorithms or software that are central to the research but not yet described in published literature, software must be made available to editors and reviewers. We strongly encourage code deposition in a community repository (e.g. GitHub). See the Nature Portfolio guidelines for submitting code & software for further information.

Data

Policy information about availability of data

All manuscripts must include a data availability statement. This statement should provide the following information, where applicable:

- Accession codes, unique identifiers, or web links for publicly available datasets
- A description of any restrictions on data availability
- For clinical datasets or third party data, please ensure that the statement adheres to our policy

The data generated in this study have been deposited into the NCBI database under accession code PRJNA873890 (https://www.ncbi.nlm.nih.gov/bioproject/PRJNA873890). These data also had been deposited in CNGB Sequence Archive (CNSA) of China National GeneBank DataBase (CNGBdb) with accession code CNP0001521 (https://db.cngb.org/search/project/CNP0001521/). The Setaria italic cv. Zhanggu reference genome (ver. 2.3) used in this study could be found in the CNSA with the accession number CNPhis0000549 (https://ftp.cngb.org/pub/CNSA/data2/CNPhis0000549/Foxtail_millet/). Other data generated in this study are provided in the Supplementary Data files.

Human research participants

Policy information about studies involving human research participants and Sex and Gender in Research.

Reporting on sex and gender	N/A
Population characteristics	N/A
Recruitment	N/A
Ethics oversight	N/A

Note that full information on the approval of the study protocol must also be provided in the manuscript.

Field-specific reporting

Please select the one below that is the best fit for your research. If you are not sure, read the appropriate sections before making your selection.

☒ Life sciences ☐ Behavioural & social sciences ☐ Ecological, evolutionary & environmental sciences

For a reference copy of the document with all sections, see nature.com/documents/nr-reporting-summary-flat.pdf

Life sciences study design

All studies must disclose on these points even when the disclosure is negative.

Sample size	We selected the cultivars which have both genotype data, root microbiota data and 12 phenotypic data from 1027 cultivars, resulting in 827 foxtail millet cultivars with RAD sequencing data, 16S rDNA amplicon sequencing data and phenotypic data. For RNA sequencing, five seedlings of foxtail millet from each treatment of the sterilized soil experiment were pooled and sequenced.
Data exclusions	We did not exclude any data from consideration.
Replication	We use 827 samples to perform GWAS on all 12 traits and root microbiota. The SNPs significantly associated with the traits and root microbiota were successfully identified based on these datasets. For the linear mixed regression model construction, we did 30 repetitions for 5-fold cross validations based on 827 samples to reduce the noise in the estimated model performance. The predictive models with the best prediction accuracy for the phenotypes using the SNP and OTU variables were obtained. For the transcriptome sequencing, we collected 5 seedlings of foxtail millet from each treatment to validate that the plant growth mechanisms mediated by microorganisms were strain-dependent. All attempts at replication were successful.
Randomization	RESPONSE: We allocated the plants into experimental groups randomly. Foxtail millet seeds were sowed in one field with 20 repeats for each cultivar. The 20 repeats from one cultivar were planted in two rows. We collected the root microbiota samples from the randomly selected three individuals for each cultivar located in the middle of rows. For the sterilized soil experiment, we randomly sowed the 10-15 germ-free HUA12 seeds into one small basin with sterilized soil and three basins for one treatment. After 14 days of inoculation, all seedlings from one treatment were collected to measure plant height and root length. In the meantime, we randomly selected 5 seedlings from each treatment to do transcriptome sequencing.
Blinding	There was some blinding as the experimenters who participated in the experimental operation and sample harvesting had no knowledge of the genetic background of the foxtail millet cultivars nor the effects of the bacterial strains on the cultivars. The experimental designation was known only to one of them.

Reporting for specific materials, systems and methods

We require information from authors about some types of materials, experimental systems and methods used in many studies. Here, indicate whether each material, system or method listed is relevant to your study. If you are not sure if a list item applies to your research, read the appropriate section before selecting a response.

Materials & experimental systems

n/a	Involved in the study	
☒	☐	Antibodies
☒	☐	Eukaryotic cell lines
☒	☐	Palaeontology and archaeology
☒	☐	Animals and other organisms
☒	☐	Clinical data
☒	☐	Dual use research of concern

Methods

n/a	Involved in the study	
☒	☐	ChIP-seq
☒	☐	Flow cytometry
☒	☐	MRI-based neuroimaging
